# Comparison of Different Carriers to Maintain a Stable Partial Nitrification Process for Low-Strength Wastewater Treatment

**DOI:** 10.3389/fbioe.2022.851565

**Published:** 2022-03-21

**Authors:** Kuo Zhang, Xinjue Li, Shou-Qing Ni, Sitong Liu

**Affiliations:** ^1^ Department of Environmental Sciences and Engineering, College of Environmental Sciences and Engineering, Peking University, Beijing, China; ^2^ Shandong Provincial Key Laboratory of Water Pollution Control and Resource Reuse, School of Environmental Science and Engineering, Shandong University, Jinan, China

**Keywords:** nitritation, anammox, composite carrier, sepiolite, nitrogen removal

## Abstract

Practical application of the partial nitritation–anaerobic ammonium oxidation (anammox) process has attracted increasing attention because of its low operational costs. However, the nitritation process, as a promising way to supply nitrite for anammox, is sensitive to the variations in substrate concentration and dissolved oxygen (DO) concentration. Therefore, a stable supply of nitrite becomes a real bottleneck in partial nitritation–anammox process, limiting their potential for application in mainstream wastewater treatment. In this study, five 18-L sequencing batch reactors were operated in parallel at room temperature (22°C ± 4°C) to explore the nitritation performance with different carrier materials, including sepiolite-nonwoven carrier (R1), zeolite-nonwoven carrier (R2), brucite-nonwoven carrier (R3), polyurethane carrier (R4), and nonwoven carrier (R5). The ammonia oxidation rate (AOR) in R1 reached the highest level of 0.174 g-N L^−1^ d^−1^ in phase II, which was 1.4-fold higher than the control reactor (R4). To guarantee a stable supply of nitrite for anammox process, the nitrite accumulation efficiency (NAE) was always higher than 77%, even though the free ammonia (FA) decreases to 0.08 mg-N/L, and the pH decreases to 6.8 ± 0.3. In phase V, the AOR in R1 reached 0.206 g-N L^−1^ d^−1^ after the DO content increase from 0.7 ± 0.3 mg/L to 1.7 ± 0.3 mg/L. The NAE in R1 was consistently higher than 68.6%, which was much higher than the other reactor systems (R2: 43.8%, R3: 46.6%, R4: 23.7%, R5: 22.7%). Analysis of 16S rRNA gene sequencing revealed that the relative abundance of *Nitrobacter* and *Nitrospira* in R1 was significantly lower than other reactors, indicating that the sepiolite carrier plays an important role in the inhibition of nitrite-oxidizing bacteria. These results indicate that the sepiolite nonwoven composite carrier can effectively improve the nitritation process, which is highly beneficial for the application of partial nitritation–anammox for mainstream wastewater treatment.

## Introduction

Nitrogen removal from wastewater is critical for the prevention of eutrophication in receiving water bodies. Anaerobic ammonium oxidation (anammox) can achieve high nitrogen removal rates without carbon consumption and therefore has received widespread attention for practical applications (Tsushima et al., 2007). However, the presence of nitrite is almost undetectable in most wastewater, especially in mainstream wastewater. Even low concentrations of nitrite in wastewater make it impossible to reach the theoretical ratio of 1:1.32(NH_4_-N:NO_2_-N) required for anammox (Strous et al., 2006; Kuenen, 2008; Zhang et al., 2018). Therefore, a stable and efficient shortcut nitrification process is a key step for the practical application of the anammox process.

Much previous research has focused on methods to establish the nitritation process, such as starvation (Nan et al., 2019) and free ammonia (FA) control (Liu et al., 2019), which are capable of achieving a high nitrite accumulation efficiency (NAE) in a short period of time. However, during actual operation, it is difficult to maintain nitritation for long time periods, especially in the case of low ammonia concentration municipal wastewater (Ge et al., 2015; Lv et al., 2020; Zhang et al., 2020). Low ammonia effluent with fluctuating dissolved oxygen (DO) content or pH causes a high growth of nitrite-oxidizing bacteria (NOB), which affects the NAE. In order to maintain the partial nitritation, it is necessary to effectively control the growth of nitrifying bacteria in the reactor system (Lv et al., 2019), with the inhibition of NOB bacterial growth requiring that the DO content in the reactor is controlled to a low range (<0.5 mg/L) (Duan et al., 2011; Tomaszewski et al., 2017; Wen et al., 2017). The lower DO content not only limits the growth of NOB, but also limits the ammonia oxidation rate (AOR) of ammonium oxidation bacteria (AOB), resulting in nitritation being unable to provide sufficient nitrite for the anaerobic ammonia oxidation process, which directly affects the anammox denitrification rate.

In order to increase AOR and NAE of nitritation, many studies have focused on the use of functional carriers, with various composite carriers developed to enhance the nitritation performance. For example, high ammonium conditions could be formed around zeolite, which are favorable for the growth of AOB (Miladinovic and Weatherley, 2008). The composite carriers based on zeolite could probably promote the metabolism of AOB and limit the activity of NOB (Lv et al., 2019). Montmorillonite could catalyze nucleic synthesis and may also play a role in the formation of primitive lipid-based membranes (Yang et al., 2021). However, these carriers are relatively high cost and exhibit weak adsorption to microorganisms, resulting in insufficient microbial enrichment (Bernal et al., 1993; Zhang et al., 2016; Yang et al., 2021). As a natural ore, sepiolite exhibits the largest specific surface area among natural nonmetallic ores, reaching 900 m^2^/g (Nagy and Bradley, 1955; Alvarez, 1984; Galán, 1996). The channel and cavity structure of sepiolite ore can absorb a large amount of water and some polar substances, providing strong adsorption capability (Preisinger, 1961; Tartaglione et al., 2008). This means that more microorganisms may be absorbed to the surface of this carrier. Furthermore, the natural weak alkalinity of sepiolite also means that this carrier may effectively improve the nitritation capacity of treatment systems (Preisinger, 1961; Galán, 1996; Kara et al., 2003). It is possible to replace zeolite with sepiolite for nitritation process.

Nonwoven carrier was often used to hold anammox microorganisms to increase the nitrogen removal rate. Combine nonwoven carrier with some functional carriers would improve the ammonia removal performance. In addition, as sepiolite is flocculent in water, combining with a traditional nonwoven carrier would prevent it from flowing out of the reactor. Thus, it is better to construct a new composite carrier that contains nonwoven fabric. In this study, in order to further verify whether sepiolite has a beneficial effect on nitritation and its potential contribution to the enrichment and growth of AOB, five 18-L sequencing batch reactors (SBRs) were operated in parallel for 151 days, at room temperature (22°C ± 4°C), to explore the effect of different functional carriers on the nitritation performance of the system, including a sepiolite-nonwoven carrier (R1), brucite nonwoven carrier (R2), zeolite nonwoven carrier (R3), polyurethane carrier (R4), and nonwoven carrier (R5). The aims of this study were (1) to explore whether the composite carrier is beneficial to the establishment of nitritation and (2) to analyze the influence of ammonia nitrogen concentration, pH, and DO on the composite carrier performance.

## Materials and Methods

### Composite Carrier Design

Five different plastic balls added with different carriers were added into five reactors to compare the nitritation performance. The plastic ball (diameter 60 mm) was made of polypropylene, which was widely used in wastewater treatment process. The composite of the carriers (Table 1) was stuffed in the plastic ball, and specific gravity close to 0.99 was ensured. For R1, the sepiolite was attached to the nonwoven fabric. For R2 and R3, the carriers, floating materials, and the nonwoven fabric are overlapped in the plastic ball, respectively. For R4 and R5, the addition of polyurethane carriers and nonwoven carriers is to further explore the compatibility of carriers. The porous structure of the plastic ball allows the substrate; oxygen could pass freely, while it could also prevent the internal carrier from escaping. The surface of composite carriers could maintain stability, which was beneficial to the stability of biofilm on the surface.

**TABLE 1:** The composition of the composite carriers.

Types	Main carriers	Total weight	Secondary carriers	Total weight	Others
I	Sepiolite (flocculent)	50 g	Nonwoven fabric (45 mm × 45 mm)	41 g	Floating material (0.08 kg/L)
II	Brucite particle (particle diameter >8 mm)	250 g	Nonwoven fabric (45 mm × 45 mm)	41 g	Floating material (0.08 kg/L)
III	Zeolite particle (particle diameter >8 mm)	250 g	Nonwoven fabric (45 mm × 45 mm)	41 g	Floating material (0.08 kg/L)
IV	Polyurethane	32 g	—	—	—
V	Nonwoven fabric (45 × 45 mm)	41 g	—	—	—

### Experimental Procedure

**FIGURE 1:**
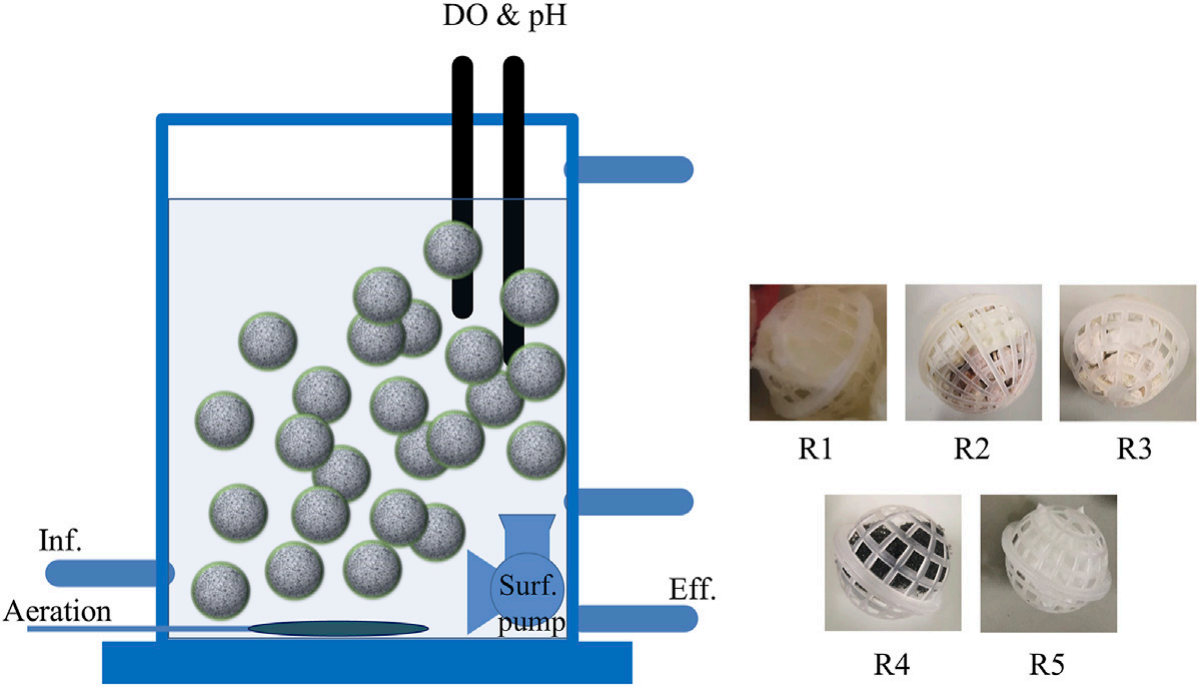
Schematic diagram of the sequencing batch reactor.

Five SBRs (Figure 1) were operated for 151 days to explore the nitritation performance. The reactor had a working volume of 18 L (15 × 30 × 40 cm), and a surf pump (2,000 L/h, 3 W, JVP110; Sensen, China) was placed in each reactor to prevent flow dead zone. This experiment can be divided into five phases (Table 2). At the beginning of phase I, the seeding sludge, obtained from Beijing Mentougou Wastewater Treatment Plant, was added with the same amount in each reactor to ensure the MLSS reached 500 mg/L. In this phase, the reactor was continuously aerated to ensure that the DO was always higher than 6 mg/L. After the precultivation, phase II (days 19–33) experiments were started with feeding synthetic wastewater into R1–R5. The hydraulic retention time (HRT) of all reactors was 6 h, and the pH and DO were maintained at 8.3 ± 0.1 and 0.7 ± 0.3 mg/L. In phase III (from days 34–83), part of the composite carriers in R1, R2, R3, and R5 was removed to ensure that the AOR of the five reactors was close. After that, the pH dropped from 8.3 to 7.5 and finally decreased to 6.8 to explore the influence of pH on composite carriers. Phase IV (days 84–115) was used to investigate the nitrite accumulation rate when the influent NH4-N concentration dropped to 40 mg/L and finally dropped to 20 mg/L. To further investigate the influence of aeration on these composite carriers, the DO concentration was increased from 0.70 ± 0.30 mg L^−1^ to 1.70 ± 0.30 mg L^−1^ on day 116. To prevent the significant reduction of effluent NH_4_-N concentration caused by the increase in DO, some of the carriers were removed, and the HRT also decreased to 2.6 h from days 116 to 151 (phase V).

**TABLE 2:** Operational parameters for phase I to V.

Phases	Time (d)	DO (mg L^−1^)	HRT(h)	Inf. pH	Inf. NH_4_-N
I (precultivation and starvation)	1–18	>6		—	—
II (ammonia oxidation rate)	19–33	0.70 ± 0.30	6	8.3 ± 0.1	60 ± 3 mg L^−1^
III (pH)	34–43	0.70 ± 0.30	4	8.3 ± 0.1	40 ± 3 mg L^−1^
	44–55	0.70 ± 0.30	4	7.5 ± 0.1	40 ± 3 mg L^−1^
	56–66	0.70 ± 0.30	4	6.8 ± 0.1	40 ± 3 mg L^−1^
	67–83	0.70 ± 0.30	4	7.5 ± 0.1	40 ± 3 mg L^−1^
IV (NH_4_-N)	84–89	0.70 ± 0.30	4	7.5 ± 0.1	40 ± 3 mg L^−1^
	90–111	0.70 ± 0.30	2	7.5 ± 0.1	20 ± 3 mg L^−1^
	112–115	0.70 ± 0.30	4	7.5 ± 0.1	40 ± 3 mg L^−1^
V (DO)	116–151	1.70 ± 0.30	2.6	7.5 ± 0.1	40 ± 3 mg L^−1^

In this experiment, all reactors were fed with synthetic wastewater prepared with tap water. NH_4_Cl was used as an ammonium source, and NaHCO_3_ was used to supply alkalinity. The synthetic wastewater also consisted of K_2_HPO_4_ 0.054 g/L and trace minerals. The trace minerals were introduced into the solution through (mg/L) FeSO_4_ 7H_2_O 9.5, CoCl_2_ 6H_2_O 0.24, CuSO_4_ 5H_2_O 0.25, H_3_BO_3_ 0.014, NaMoO_4_ 2H_2_O 0.22, NiCl_2_ 6H_2_O 0.19, and MnCl_2_ 4H_2_O 0.99 (Yin et al., 2016; Graaf et al., 2017). One percent sulfuric acid and saturated sodium carbonate solution were used to adjust the pH in the reactors.

### Measurement and Analysis

The concentrations of NH_4_^+^-N, NO_2_^−^-N, NO_3_^—^N, and MLSS were measured according to standard methods (APHA, 2005). DO and pH were measured using online probes (Leici, China). FA was calculated (Anthonisen et al., 1976) as follows:

$${\text{C}}_{\text{FA}}=\frac{17}{14}\frac{{\text{C}}_{\text{total,}\;\text{AMMONIUM}}\times 10^{\text{pH}}}{{\text{e}}^{6344/\left(273+\text{T}\right)}+10^{\text{pH}}}$$ (1)

AOR was calculated a followss:

$$\text{AOR}\left(\text{g}-\text{N}\cdot {\text{L}}^{-1}\cdot {\text{d}}^{-1}\right)=\frac{\left({\text{NH}}_{4}^{+}{-\text{N}}_{\text{Inf}}{-\text{NH}}_{4}^{+}{-\text{N}}_{\text{Eff}}\right)\times 24}{1000\cdot \text{HRT}}$$ (2)

NAE was calculated as follows:

$$\text{NAE}=\frac{{[\text{NO}}_{2}^{-}-\text{N}]}{{[\text{NO}}_{2}^{-}{-\text{N}]+[\text{NO}}_{3}^{-}-\text{N}]}\times 100\%$$ (3)

### Microbial Community Analysis

Sludge sample P1 was collected from the seeding sludge. Other samples were collected from each reactor (three samples were taken from the top, middle, and bottom of the reactor, and then they were mixed as one sample) on days 30 and 151. The sample points in R1, R2, and R3 are on the surface of the functional carrier (sepiolite, zeolite, and brucite). DNA extraction was performed immediately after each sampling was completed and then stored in the refrigerator (−20°C).

Microbial community was analyzed as the following procedure. Microbial DNA was extracted using the FastDNA @ SPIN Kit for soil (MP Biomedicals) flowing the manufacturer’s introduction. The bacterial 16SrRNA gene was amplified using the primer pair 338F/806R targeting the V4 region (Bhattacharjee et al., 2017). High-throughput sequencing was conducted at Majorbio Co., Ltd. (Shanghai, China) using the Illumina MiSeq platform (Loman et al., 2012). The Circos diagram was completed using I-Sanger based on 16S rRNA sequencing (http://www.i-sanger.com/).

## Results

### Reactor Performance

**FIGURE 2:**
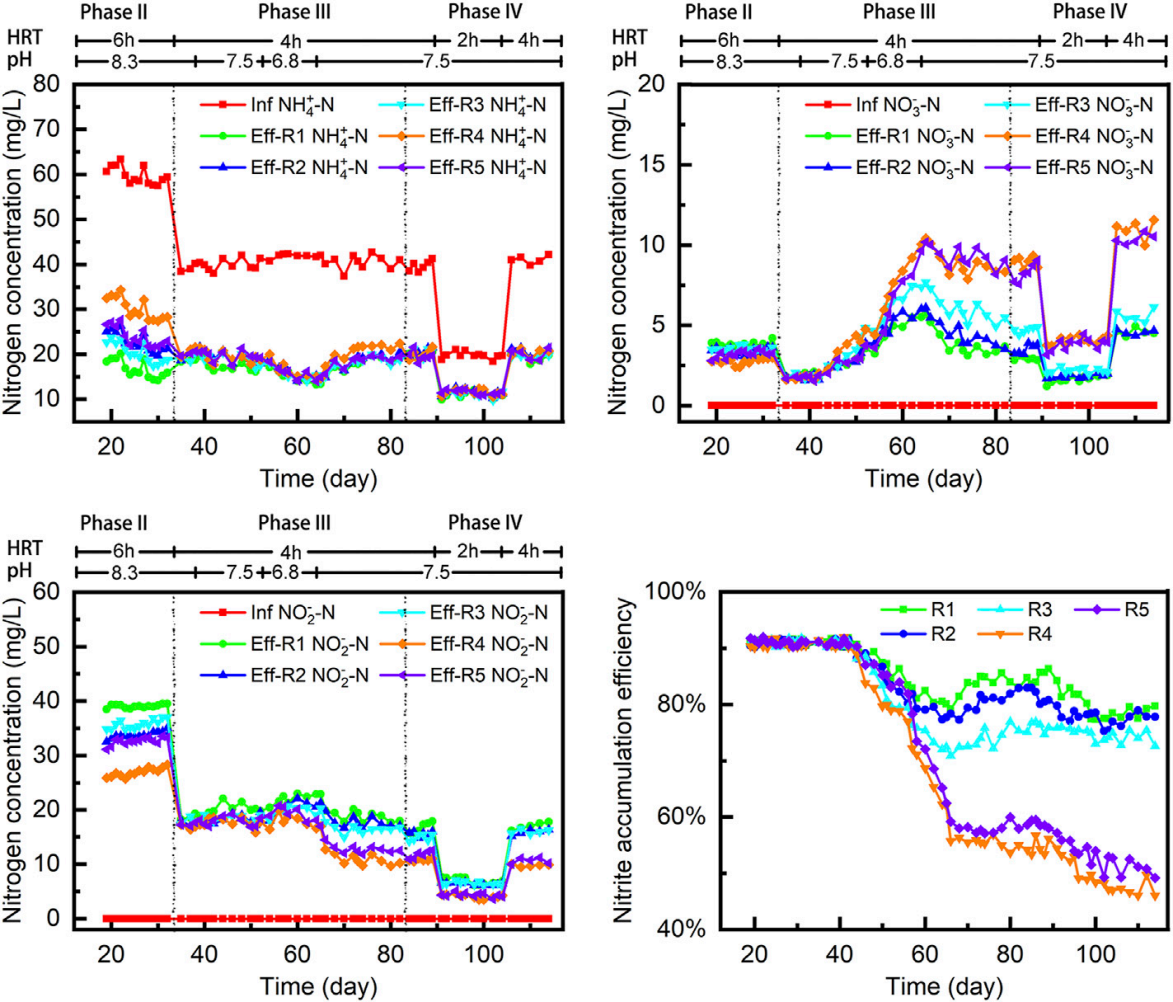
Performance of these reactors during first 115 days’ operation (phases I–IV).

Variations in the nitrogen concentration during the first 34 days of operation are shown in Figure 2. After prestarvation, greater than 90% of ammonia was converted to nitrite in each group, under pH 8.3 conditions, indicating that all the reactors achieved a good nitritation performance. Nitritation was established after phase II. Among the five reactor systems, the AOR in R4 was lowest at 0.124 g-N L^−1^ d^−1^, with the effluent NH4-N concentration reducing to only 30 mg/L with a 6-h HRT. Reactor R5 contained a nonwoven control carrier and maintained an average effluent NH_4_-N concentration of lower than 24 mg/L. Similarly, the average effluent NH_4_-N concentrations in R2 and R3 were 22.6 and 20.9 mg/L, respectively. The AORs in R2 and R3 were 0.152 and 0.162 g-N L^−1^ d^−1^, respectively, which was closed to R5. Compared with other nonwoven fabric combinations, the average effluent NH_4_-N concentration in R1, which contained sepiolite, decreased to 16.7 mg/L. After 32 days of operation, the AOR in R1 reached 0.174 g-N L^−1^ d^−1^, which was 1.1- to 1.4-fold higher than the other systems.

In the beginning of phase III, to make sure the AOR in each reactor was a closed part of the carriers removed in R1, R2, R3, and R5. Meanwhile, the NAE of each reactor was not affected by the lower influent NH_4_-N concentration and consistently remained at approximately 91% from days 34 to 43. However, although the effluent NH_4_-N concentration in each reactor was maintained at between 16 and 19 mg/L, the NAE gradually decreased with a reduction in pH from 8.3 to 7.5. This trend was most obvious in reactors R4 and R5, with the NAE decreasing to 55.2% and 59.3%, respectively, when the pH reduced to 6.8. In contrast, the reactors containing composite carriers (R1, R2, R3) performed better than the control reactors (R4 and R5). From days 56 to 66, the NAE in R1 was consistently higher than 79%, and when the pH increased back to 7.5 from days 67 to 82, the NAE in R1 increased to 83.2%. The effluent NO_3_-N concentrations in R2 and R3 (3.74 and 4.92 mg/L) were slightly higher than R1 (3.19 mg/L). At the end of phase III, the NAE in R2 and R3 reduced to 80.7% and 75.3%, respectively.

At the beginning of phase IV, part of the carrier was removed from each reactor to maintain a consistent AOR. After the influent NH_4_-N concentration decreased to 20 mg/L, the effluent NH_4_-N concentration also decreased to 11 mg/L, with the HRT shortened to 2 h. Because of the NAE in R4 and R5 being much lower than the other reactors, the average effluent NO_3_-N concentration reached 4.3 and 4.1 mg/L, respectively, from days 91 to 111. For R1 and R2 reactors containing the sepiolite or brucite carriers, even with a reduction in effluent NO_2_-N concentration to 6.4 mg/L and 6.2 mg/L, respectively, the total NAE value remained consistently higher than 78%. On day 111, the effluent NO_2_-N concentration in R1 also reached 6.9 mg/L, which was 1.61- and 1.71-fold higher than R4 and R5, respectively.

### The Influence of DO Concentration

**FIGURE 3:**
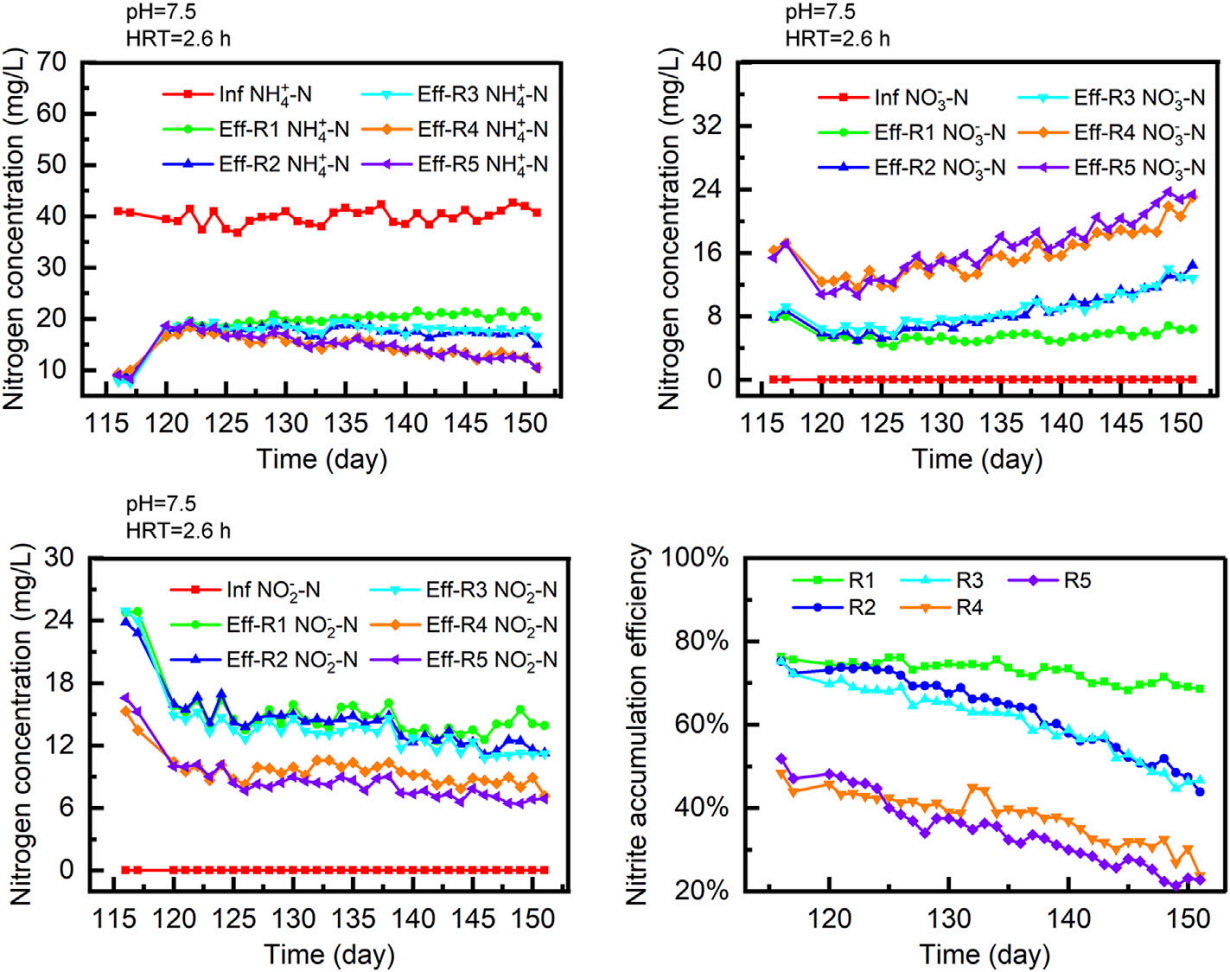
The performance of each reactor in phase V.

As shown in Figure 3, variation in the DO content significantly affected the nitritation performance of the system. From days 116 to 117, the DO content increased to 1.7 mg/L, causing the AOR in each reactor to increase by 45% to 55%. After the HRT was reduced to 2.6 h, the effluent NH_4_-N concentration increased from 9 to 18 mg/L. It was observed that the NAE in reactors R4 and R5 continually decreased, finally reducing to 23.7% and 22.7%, respectively. After 35 days of operation, the effluent NO_3_-N concentration in R5 increased from 10.7 to 23.8 mg/L.

From days 116 to 130, the NAE in R2 and R3 was maintained at approximately 69% and 65%, respectively. However, the effluent NO_3_-N increased after day 130. In the following 21 days of operation, the effluent NO_3_-N content increased to 13.5 ± 0.9 and 13.3 ± 0.7 mg/L, respectively. The NAE of reactors R2 and R3 finally reduced to 43.8% and 46.6% on day 151, whereas the NAE of R1 was consistently higher than 68.6% in this phase. Although the effluent NO_2_-N concentration exhibited a small decline from days 116 to 130, the effluent NO_3_-N content remained less than 6.8 mg/L in the following days.

### Microbial Community Analysis

**FIGURE 4:**
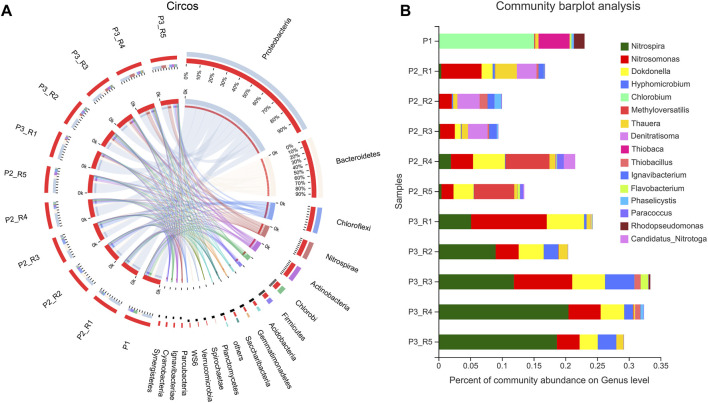
**(A)** Distribution of phylum on different carriers based on the taxonomy annotations from NCBI-NR database. **(B)** Percent abundances of the quantitatively prominent bacteria by genus **(B)** in these samples.

To investigate the community discrepancy between these composite carrier, 16S rRNA gene sequencing was conducted. The sequencing depth of each sample was more than 42,900 reads. Rarefaction curves (Supplementary Figure S1) suggested that the sampling of bacterial richness was complete, and the entire coverage index of >0.99 also supported that the coverage degree of the MiSeq sequencing was high. As shown in Figure 4, Proteobacteria and Bacteroidetes were the dominant bacterial phyla, which account for more than 50% in each sample point. Compared with the seeding sludge (P1), Chloroflexi was greatly increased after 30 days’ operation. In R2 and R3, the relative abundance of Chloroflexi increased from lower than 1‰ to 19.3% and 18.6%, respectively. Besides, the Nitrospirae became the third dominant phylum after DO rise to 1.7 ± 0.3 mg/L. It could be found that the total reads increased from 2.0% to 20.5% in R4 and from 0.5% to 18.7% in R5.

The analytical results at the genus level showed that the relative abundance of the typical AOB bacteria *Nitrosomonas* was less than 1‰ in the seeding sludge (P1). However, after starvation and aeration, the abundance of the AOB flora of R1-R5 began to increase. The relative abundance of (*Nitrosomonas*) in R1 was the highest, reaching 6.4%, which was significantly higher than other control reactors (2% of R2, R3), 2.3% of R4 and 1.9% of R5). Although almost no NOB bacteria were detected in each reactor on day 30, the NOB bacteria dominated by *Nitrospira* were significantly increased after phase V. Under 1.7 ± 0.3 mg/L DO concentration, the relative abundance of *Nitrospira* reached 20.5% in R4 and 18.7% in R5. In contrast, the relative abundance of *Nitrosomonas* was only increased from 3.5% to 5.1% in R4 and 1.9% to 3.6% in R5. In R2 and R3, the relative abundance of *Nitrosomonas* increased slightly to 3.6% and 9.2%, but the relative abundance of *Nitrospira* also increased to 9.0% and 11.9%, respectively. Compared with other reactors, the total effective reads of *Nitrosomonas* increased to 12.0%, whereas the amount of *Nitrospira* was less than 5.2% in R1.

## Discussion

A main challenge in the mainstream application of partial nitritation is the low ammonia concentration and pH of wastewater. Under lower ammonia concentration or pH conditions, NOB cannot be suppressed, resulting in the conversion of large amounts of accumulated nitrite to nitrate. Therefore, it is particularly important to explore new types of composite carrier that support efficient and stable nitritation performance under these conditions. In this study, the necessity for a composite carrier to establish the nitritation process was verified, and the newly developed sepiolite-nonwoven carrier was found to achieve a higher AOR and NAE than other conventional carriers, with the AOR in R1 1.4-fold higher than R4 in phase II. It can be concluded that under the same carrier dosing conditions, the reactors containing nonwoven fabric exhibited stronger microbial adhesion than the polyurethane system. On the other hand, the ammonia oxidation performance of R2, R3, and R5 indicated that even if 250 g of ore carriers was added in R2 and R3, the AOR was slight increased to no more than 12%. This means the conventional ore carrier was hardly to enrich the microorganisms. To increase the MLSS in a reactor based on composite carrier is the key to improving its application.

The AOR in R1 was 174 mg NH_4_-N d^−1^ L^−1^, which was 15% and 8% higher than in R2 and R3, respectively. The 16S sequencing results also indicated that the relative abundance of AOR in R1 reached 6.4%, which was more than threefold higher than other nonwoven carrier reactors (2.0% in R2, 2.0% in R3, and 1.9% in R5). These results suggest that microorganisms had a higher affinity to sepiolite, indicating that sepiolite is capable of better colony enrichment than the other tested carriers (brucite or zeolite). The morphological characteristics of carrier material in R1 show that sepiolite exists in the form of a flocculent in water, naturally adsorbing on to the surface of nonwoven fabric (Figure 1). Furthermore, sepiolite provides a unique pore structure, with a large specific surface area, and thus, the average effluent NH_4_-N concentration in R1 was much lower than the other reactors. Therefore, it can be concluded that the composite carrier based on sepiolite could achieve a higher AOR and provide better conditions for the enrichment of AOB bacteria.

Some researchers indicated that a higher FA concentration (>0.1 mg/L) significantly benefits the nitritation process because of its ability to inhibit the growth of NOB (Cho et al., 2011; Wen et al., 2017; Liu et al., 2019). In the present study, the FA concentration in the reactor was maintained at greater than 2 mg/L NH_3_-N in phase II by increasing the pH to 8.3. Therefore, the reactors achieved a better NAE (>90%) after the starvation phase. However, when the pH was reduced from 8.3 to 7.5 and finally to 6.8 during phase III, the FA concentration also reduced from 2 to 0.4 mg/L and finally to 0.06 mg/L. The effluent NO_2_-N concentration shows that the NAE of R4 and R5 continuously decreased after the pH was reduced. When the pH was reduced to 6.8, the NAE in R4 and R5 decreased to 55.7% and 59.1%, respectively, with the reduction in NAE continuing even after the pH was increased back to 7.5. Results indicate that the conventional carriers play only a partial role in the enrichment of microorganisms, whereas the reduction in FA concentration directly affects the entire nitritation process. The response of R1 and R2 to pH fluctuations was notably lower than that of R4 or R5, which may be due to sepiolite and brucite being weakly alkaline in water, resulting in a solution pH (after sepiolite and brucite were placed in the reactor for 48 h without seeding sludge) of 7.95 and 8.23, respectively. In combination with the finding that in phase III the NAE consistently remained >79% and 77% in R1 and R2, respectively, it can be inferred that the carrier (which releases trace levels of alkalinity) forms a transition layer on its surface, restricting the growth of NOB on its surface. Otherwise, because of the slow alkalinity release rate, no change was observed in the macroscopic pH value of the solution inside the reactor. Thus, the microorganisms in R1 and R2 were not sensitive to fluctuation in pH. Sepiolite presents in the form of a flocculent in water, becoming evenly distributed on the surface of nonwoven fabric due to strong adsorption, while the brucite is massive. Therefore, both the capacity of brucite for bacterial colonization and the binding properties of nonwoven fabrics are inferior to those of sepiolite. Furthermore, as gaps exist in the contact layer between brucite and the nonwoven fabric, the environmental conditions in this area are similar to those of R5, resulting in the NAE of R2 being lower than R1. The reduction in NH_4_-N concentration had little effect on reactors containing the composite carrier (R1, R2, and R3). The zeolite carrier that was used in R3 is considered to have the characteristics of easily absorbing ammonia, according to previously reported studies, allowing it to achieve a better nitritation performance under low NH_4_-N concentration conditions. Consequently, the experimental results for phases II and III show that a change in pH more obviously affects the NAE.

DO is another parameter that affects the nitritation performance, with fluctuation in its value directly affecting the AOR and NAE of the reactor. Higher DO conditions not only help to increase the AOR, but also contribute to the enrichment of NOB bacteria, directly leading to an increase in effluent NO_3_-N concentration. Some studies have reported that a lower DO concentration (<0.5 mg/L) could inhibit the growth of NOB bacteria. However, under low DO concentration conditions, the activities of AOB are also significantly reduced. In the present study, as the DO concentration was increased from 0.7 to 1.7 mg/L, the effluent NH_4_-N concentration decreased to 5.9 to 7.7 mg/L. The AOR in each reactor increased by 45% to 55%. Unfortunately, the effluent NO_3_-N concentration in R4 R5 also gradually increased in accordance with the DO concentration. These results verify that higher DO conditions could increase the nitrification rate. The 16S sequencing results also show that the abundance of NOB bacteria such as *Nitrospira* increased to 20.5% in R4 and 18.7% R5, after 35 days of operation. This illustrates that it is difficult for conventional biological carriers to maintain the nitritation process under higher DO conditions. The special AOB and NOB activities both increased after the DO content was increased. With composite carriers (R1, R2, and R3), the NAE was stable in the first 10 days after the DO content increased. Nevertheless, the NAE of R2 and R3 began to decline from days 130 to 151. The relative abundance of *Nitrospira* increased to 9.0% and 11.9% in R2 and R3, respectively, which further supported the finding that higher DO concentration conditions lead to the growth of NOB bacteria. In contrast, the relative abundance of *Nitrospira* in R1 was less than 5.2%, which was much lower than in R2 or R3. Compared with R1 and R2, both sepiolite and brucite were found to release alkalinity in this study, with the difference that sepiolite functions as a flocculent, containing void internal channels, and can be wrapped on the surface of the nonwoven fabric. This difference may account for R1 achieving a higher NAE under high DO conditions. Because of sepiolite being wrapped around the nonwoven fabric, the biofilm structure formed by the composite carrier was much thicker than in the other reactors. The external bacteria reduce the impact of DO on the internal bacteria, resulting in a lower relative abundance of NOB than other groups. Furthermore, the total effective reads of *Nitrosomonas* increased to 12.0% in R1, exhibiting a much higher increase than in R2 or R3, supporting the theory that the porous structure of sepiolite creates a three-dimensional space for bacteria to help avoid the impact of DO. Therefore, it can be concluded that sepiolite can be fused with nonwoven fabric to form an effective composite carrier for future application, capable of achieving stable nitritation performance.

These results suggest that this novel composite carrier is effective and superior to the conventional carriers used for the nitritation process. Using this novel carrier, a wastewater treatment device with a processing capacity of 15 m^3^ d^−1^ was implemented and has been stably operated for nearly 4 months, achieving an average NAR of nearly 80% under lower influent NH_4_-N concentration conditions (24–45 mg/L), with the treated effluent providing sufficient nitrite for the anammox process.

## Conclusion

Ensuring stable nitritation, with tolerance to the shock caused by varying influent conditions, is a key factor in successful anammox treatment processes. The results of this study verify the high performance of sepiolite-nonwoven composite carriers in the establishment of nitritation processes. The use of sepiolite-nonwoven fabrics as a functional carrier provides various advantages, significantly improving the capacity of the reactor to resist pH or DO fluctuations. This new composite carrier can also supply nitrite for the anammox process under high DO conditions (>1.7 mg/L). The analysis results of 16S sequencing showed that the sepiolite nonwoven fabric carrier can restrict the growth of NOB to a certain extent. Overall, this composite carrier provides a useful strategy for the operation of the mainstream partial nitritation–anammox processes, with good potential for widespread practical application.

## Data Availability

The datasets presented in this study can be found in online repositories. The names of the repository/repositories and accession number(s) can be found in the article/Supplementary Material.
