# Supplementary material for: Comparison of Different Carriers to Maintain a Stable Partial Nitrification Process for Low-Strength Wastewater Treatment
Source: Front Bioeng Biotechnol. 2022 Mar 21;10:851565. doi: 10.3389/fbioe.2022.851565 (PMC8979113; doi:10.3389/fbioe.2022.851565)
Supplement: Supplementary file 1 [file DataSheet1.PDF]

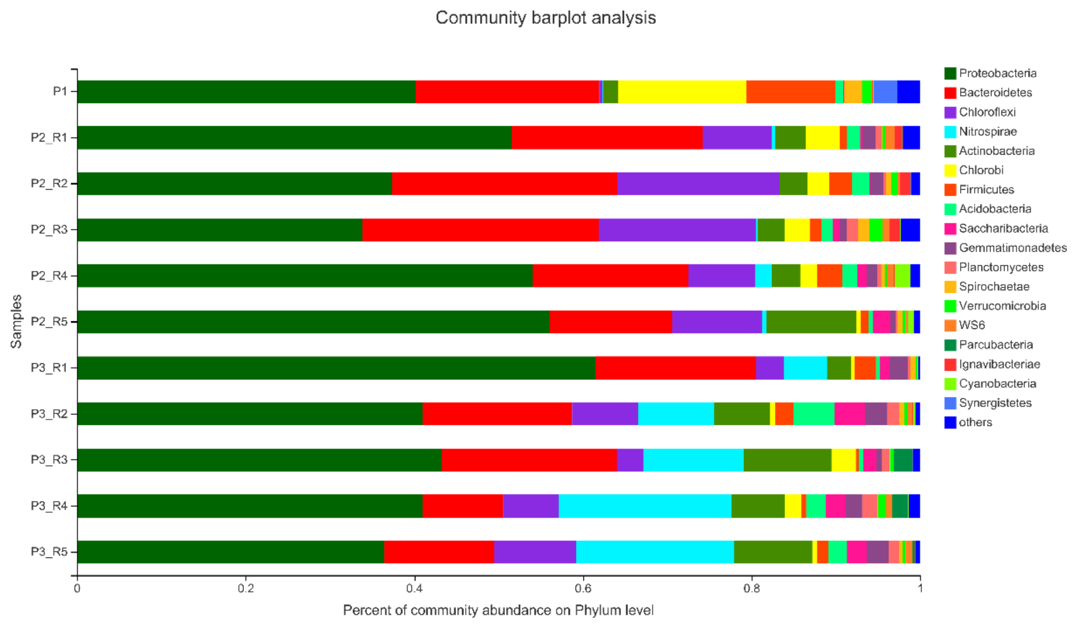


Fig. S1. Percent abundances of the quantitatively prominent bacteria by phylum in eleven samples

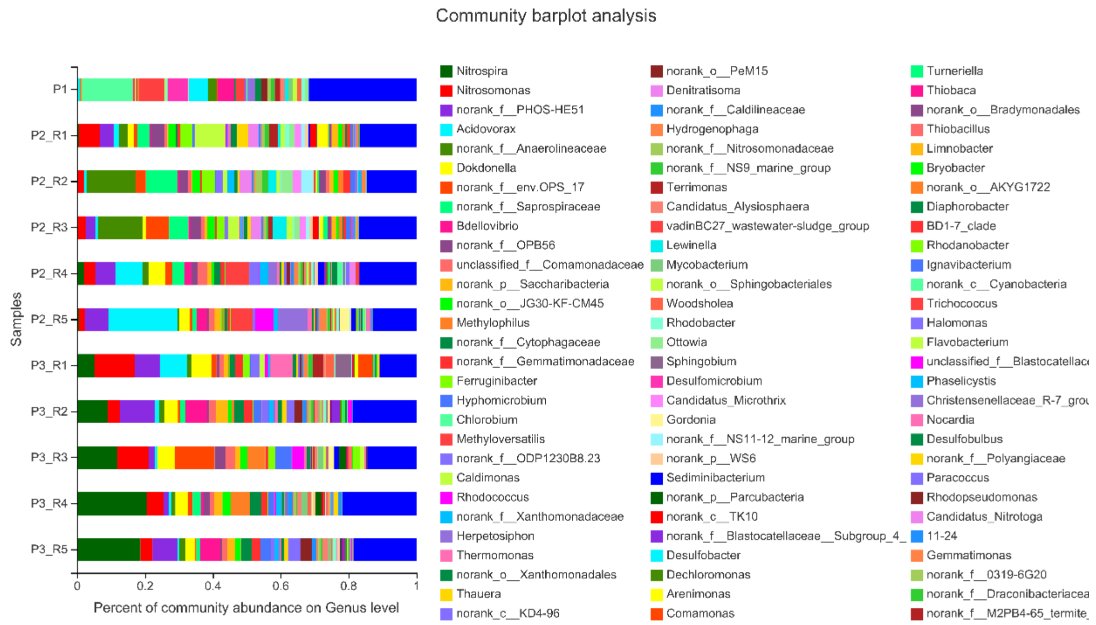


Fig. S2. Percent abundances of the quantitatively prominent bacteria by genus in eleven samples

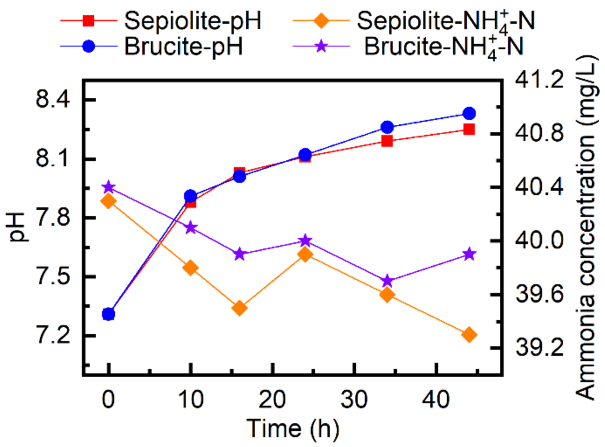


Fig. S3. The changes of pH and ammonia concentration after 44 hours.

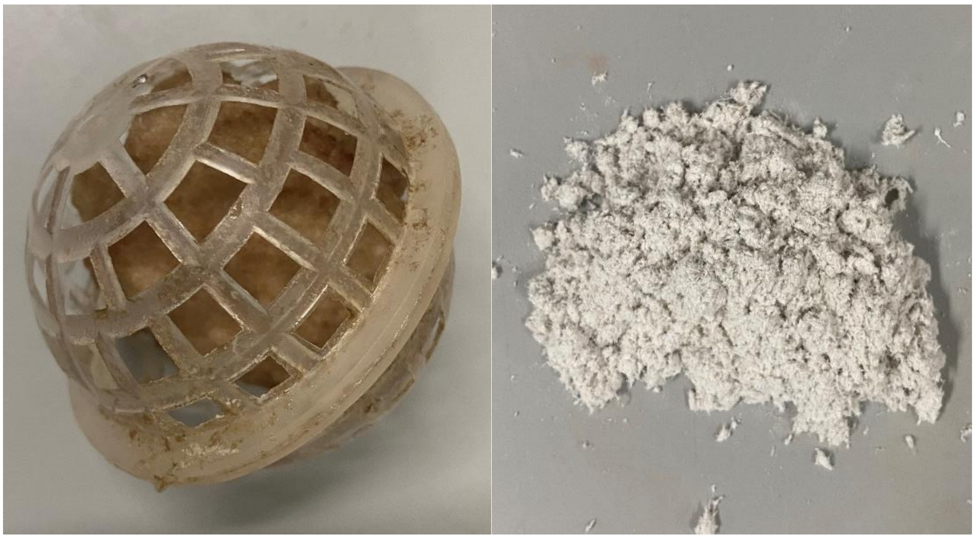

Fig. S4. The morphology characteristics of the sepiolite

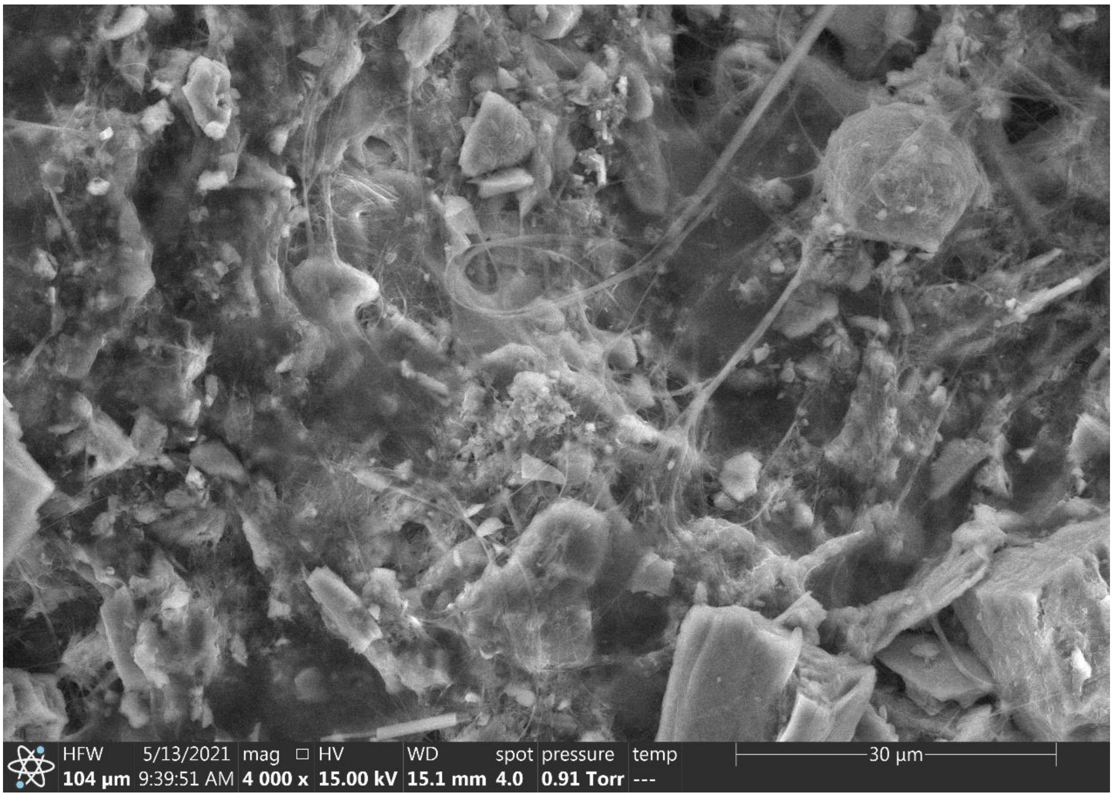

Fig. S5. SEM micrographs of sepiolite.

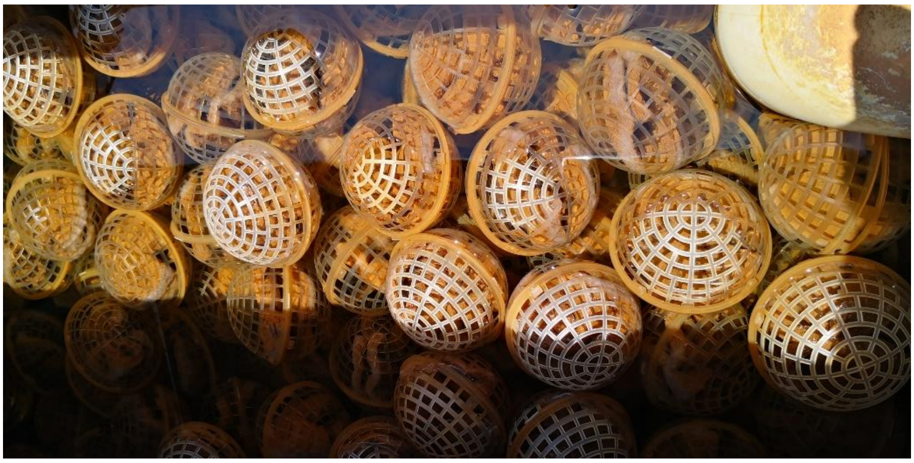

Fig. S6. The morphology of the composite carriers in the wastewater treatment device
